# Azygos vein stenting as an alternate route for inferior vena cava obstruction

**DOI:** 10.1016/j.jvscit.2023.101351

**Published:** 2023-10-23

**Authors:** Christopher Montoya, Camilo Polanía-Sandoval, Jose I. Almeida

**Affiliations:** Division of Vascular Surgery, DeWitt Daughtry Family Department of Surgery, University of Miami, Leonard M. Miller School of Medicine, Miami, FL

**Keywords:** Azygos vein, Case report, Chronic Venous Insufficiency, Stenting

## Abstract

Chronic venous insufficiency, caused by inferior vena cava occlusion, can lead to thromboembolic complications and tissue loss. We present two cases of azygos vein stenting (AVS) in which vena cava recanalization techniques were exhausted. In the first case, the left iliac vein and vena cava were recanalized and stented; however, the right iliac vein had been previously resected and required AVS. Conventional recanalization attempts from the right and left iliac systems failed in the second patient with congenital inferior vena cava occlusion; therefore, AVS was chosen to establish in-line drainage

Chronic venous insufficiency (CVI) is a common condition that negatively affects patients’ quality of life.^1^ It results from venous valve reflux or proximal venous flow obstruction, including inferior vena cava (IVC) occlusion (IVCO) caused by extrinsic compression or adjacent malignancy. The treatment options for IVCO-related CVI include stent implantation, angioplasty, or bypass surgery.^2^^,^^3^ We present two cases of patients with CVI who were successfully treated with endovascular azygos vein stenting (AVS). Both patients provided written informed consent for the report of their case details and imaging studies.

## Case report

### Patient 1

**Fig 1:**
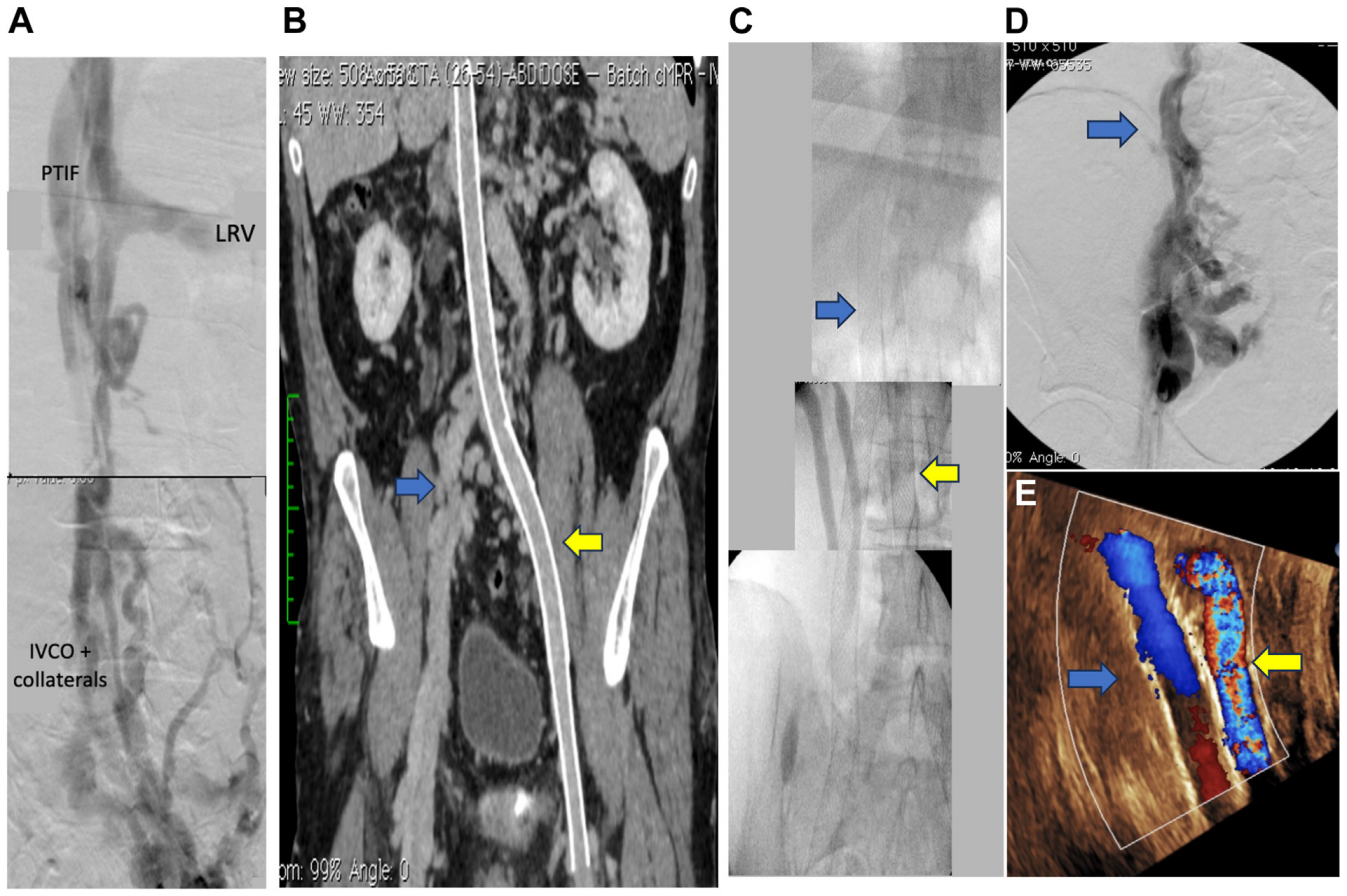
**A,** Angiogram showing post-thrombotic left lower extremity with iliac vein occlusion and caval outflow. **B,** Computed tomography scan of left inferior vena cava occlusion (IVCO) stenting. The *blue arrow* indicates right common iliac vein occlusion with pelvic collateral veins; and the *yellow arrow* shows left iliocaval stent placement. **C,** The *blue arrow* shows the stented right common femoral vein and azygos vein, with drainage into the superior vena cava (SVC); the *yellow arrow* depicts the stented left common femoral vein and iliac vein, with drainage into the suprahepatic inferior vena cava. **D,** Venogram showing right-sided pelvic collateral vessels draining into the azygous vein before azygos vein stenting (AVS). **E,** Duplex ultrasound (image rotated) at 12 years showing patent right azygos (*blue arrow*) and left caval (*yellow arrow*) stents. *LRV,* Left renal vein; *PTIF,* post-thrombotic intraluminal fibrosis.

**Fig 2:**
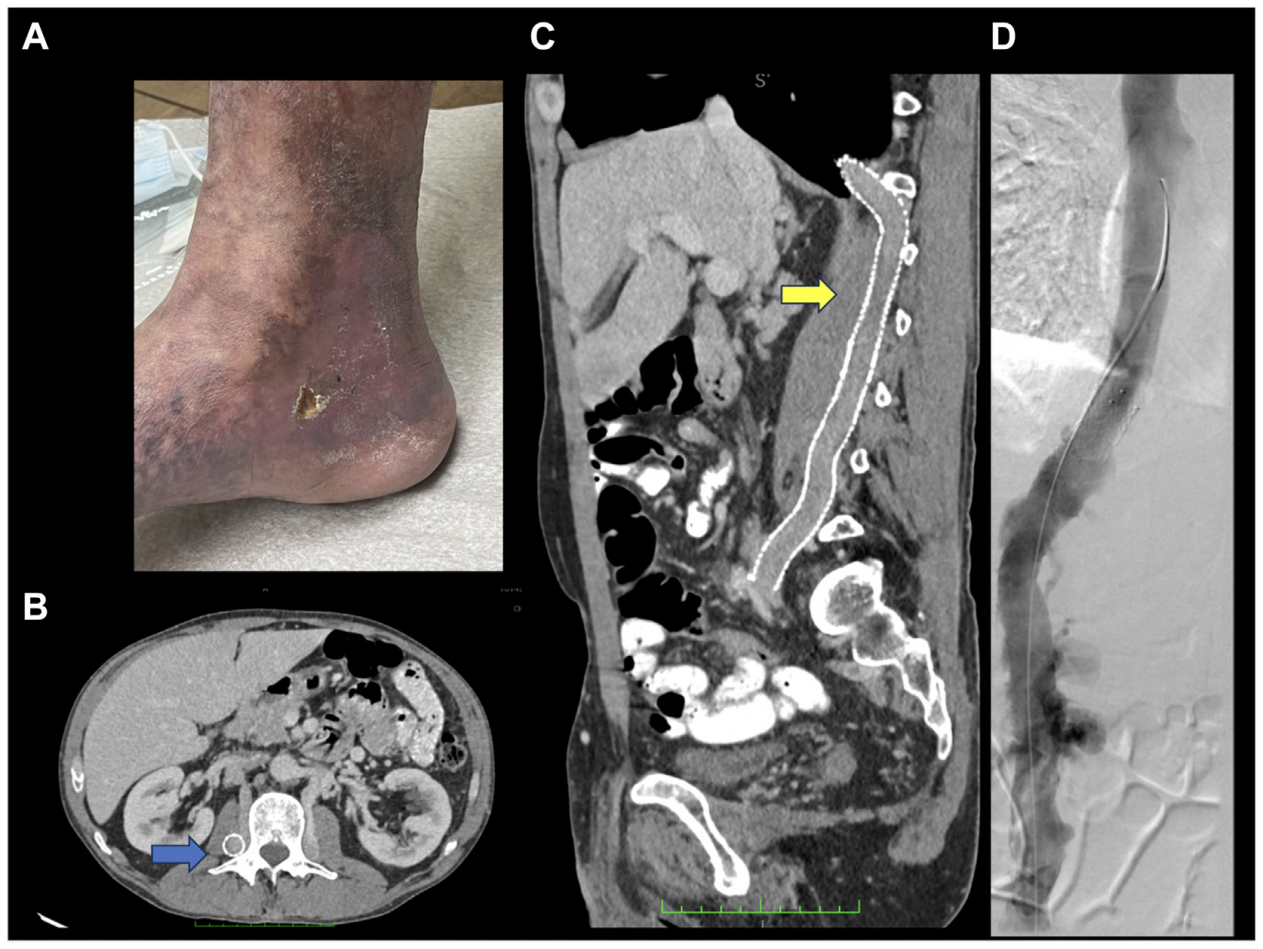
**A,** Photograph showing a venous ulcer at the right medial malleolus. **B,** Axial computed tomography scan showing azygous stent (*blue arrow*). **C,** Coronal computed tomography scan showing azygos stent draining into suprahepatic vena cava (*yellow arrow*). **D,** Venogram of the azygos vein after stenting.

A 70-year-old man presented to us with a history of recurrent deep vein thrombosis (DVT) and venous leg ulcers (VLUs) in both legs for >20 years due to a childhood cancer surgery complication. He had been managed conservatively by us for 8 years with compression hosiery and warfarin. In 2012, we offered the patient endovascular reconstruction. We were unable to reconstruct the right iliofemoral system, but we successfully stented the left side from the common femoral vein to the intrahepatic vena cava. In 2013, we did another attempt, with the intent of crossing the right retroperitoneal collateral vessels to gain access to the contralateral left iliac vein stent. This was unsuccessful; however, from the same right femoral vein access, we were able to navigate and cross the tortuous azygos vein outflow with a stiff angled Glidewire (Terumo Interventional Systems) supported with the TriForce peripheral crossing set (Cook Medical, Inc) and enter the right atrium (Fig 1). The TriForce set was advanced using an 8F × 35-cm Terumo sheath (Terumo Interventional Systems) telescoped through our primary groin access sheath (11F × 11 cm). Systemic anticoagulation was administered at this point. Serial balloon dilation of the entire tract was performed carefully, initiating with a 5-mm balloon and completing with a 14-mm balloon. Intravascular ultrasound was used for stent diameter sizing, stent length determination, and choosing the proximal and distal landing zones. Three 14-mm Wallstents (Boston Scientific) were stacked from the proximal azygos system down to the common femoral vein, creating a femoral-to-superior vena cava (SVC) connection. After two procedures, the final stent stacks consisted of left femoral–IVC and right femoral–SVC endovascular reconstruction (Fig 2). He was prescribed rivaroxaban postoperatively. His reconstruction has been patent for 12 years, and he has also been free of DVT and VLUs since his treatment.

### Patient 2

**Fig 3:**
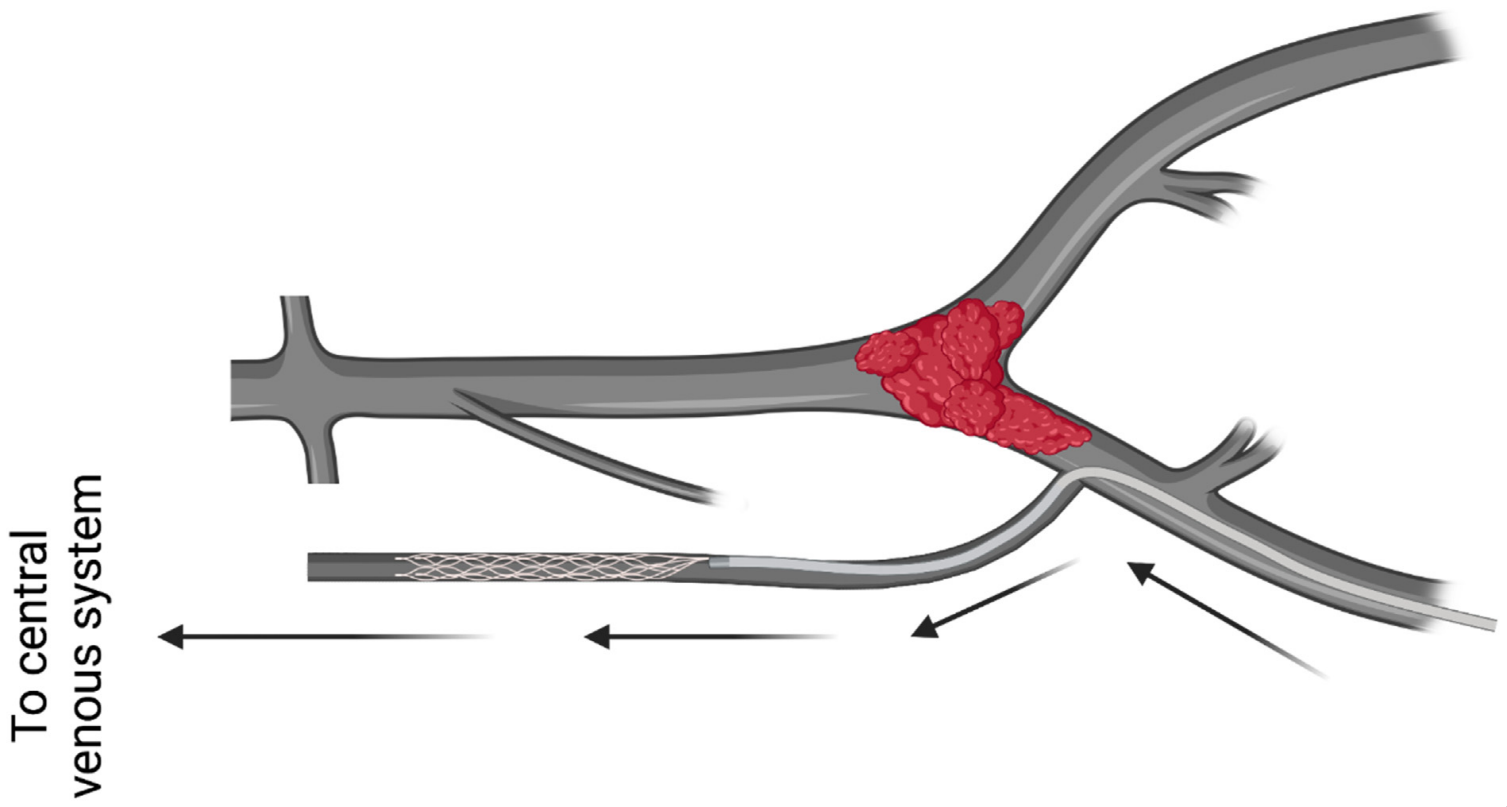
Schematic representation of inferior vena cava occlusion (IVCO) and azygos vein stenting (AVS). Created with biorender.com.

Patient 2 is a 51-year-old man with a history of recurrent VLUs at the right medial malleolus and multiple DVTs in both legs during a 20-year period. Previous treatments at another facility included right great saphenous vein ablation and failed recanalization of the bilateral iliocaval veins. In 2023, we performed endovascular revascularization using bilateral femoral vein access. Venography showed patent bilateral iliofemoral veins with no contrast filling in the IVC. The primary outflow on the right side was through a highly stenotic azygos vein; on the left side, it was through the ascending lumbar veins. Venography via right internal jugular vein access confirmed SVC patency and an absent infrahepatic IVC, suggesting this was IVC atresia (IVCA). We were unable to find an entry point to the IVC from below and confirmed that the main right iliofemoral outflow was through the azygos vein. We successfully crossed the azygos vein using a stiff angled Glidewire, supported by the TriForce peripheral crossing set (Cook Medical Inc). The TriForce was advanced using an 8F × 35-cm sheath (Terumo Interventional Systems) telescoped through our primary groin access sheath (11F × 11 cm). Systemic anticoagulation was administered at this point. After entering a channel connected to the intrahepatic segment of the IVC, serial balloon venoplasty and stenting were performed. A Zilver Vena stent stack (Cook Medical Inc), 14 × 140 mm, was placed in the azygos vein with proximal landing in the suprahepatic IVC. This stent has the lowest chronic outward force of the available laser cut nitinol stents, making it suitable for the paraspinal space. Intravascular ultrasound was used for stent diameter sizing, stent length determination, and choosing the proximal and distal landing zones. Completion venography showed wide patency of the stented azygos vein with runoff into the right atrium (Fig 3). He was prescribed apixaban postoperatively, and his only complaint was meralgia paresthetica that resolved after 3 months. The meralgia paresthetica was probably due to stent compression or injury at the axis site in the mid-thigh. The stent has been patent for 3 months, and he has been free of DVT and VLUs since his treatment.

## Discussion

The azygos system is normally a paired paravertebral venous pathway in the posterior thorax. The junction of the right ascending lumbar and subcostal veins forms the azygos, which ascends and drains to the SVC. Important tributaries of the azygos include the hemiazygos vein on the left side and the intercostal, esophageal, and lumbar veins. Collateral pathways play a crucial role in maintaining proper drainage from the legs and pelvis through the deep, median, portal, and superficial pathways.^4^, ^5^, ^6^

Malformations of the IVC include IVCA, a left IVC, double IVC, azygos continuation of the IVC, a circumaortic left renal vein, a retroaortic left renal vein, a double IVC with a retroaortic right renal vein and hemiazygos continuation, a double IVC with retroaortic left renal vein and azygos continuation, a circumcaval ureter, and isolated accessory hemiazygos vein.^5^^,^^7^ IVCA has a prevalence of 0.0005% to 1%^6^ and occurs due to underdevelopment of the posterior and supracardinal veins.^7^ In our second patient, ICVA was likely the cause. Reports of patients with IVCA and DVT have reported successful management with anticoagulation and compression hosiery, without DVT recurrence.^6^^,^^8^ Among 62 reported cases, most patients were treated similarly, with some receiving thrombolysis before anticoagulation and a few requiring bypass surgery.^6^

IVCO treatment typically involves stent placement in veins such as the common femoral, external iliac, and common iliac veins and the IVC, with a high success rate.^9^ Therefore, when navigating the retroperitoneal lumbar to azygos connections in the context of chronic IVCO, the technical aspects are similar to performing conventional iliac vein recanalization. However, establishing the drainage of the azygos to the SVC is crucial. Angled Glidewires, support catheters, and guide sheaths should be used. Complications such as traversing the spinal canal or disrupting important collateral vessels can occur. Thus, gentle balloon dilations, multiple orthogonal views, and the liberal use of "puff" venography is advised to ensure that the wires are in the proper location. In the case of perforation, a coda balloon might be helpful. After stent placement in patients with post-thrombotic syndrome, lifelong anticoagulation is needed with direct oral anticoagulation drugs.

In our literature search, we found no reports of AVS specifically for thrombotic IVCO. However, the use of azygos stenting to treat other causes is summarized in Table I.^10^, ^11^, ^12^ Other case reports also describe interventional treatment of azygos vein aneurysms resulting from different causes.^13^ Our cases contribute to the literature regarding the effectiveness of this procedure, with, to the best of our knowledge, our first case the first reported in the context of thrombotic IVCO. Also, we observed clinical improvement at follow-up (Table II), without the complications reported in the literature. These cases underscore the significance of the collateral circulation and emphasize the consideration of AVS as an alternative route for patients with IVCA and post-thrombotic IVCO. Larger studies are required for a deeper understanding of the long-term outcomes.^10^

**Table I:** Summary of reported cases of endovascular azygos vein stenting

Investigator	Age, years	Sex	Comorbidities	Indication	Procedure	Stent type	Follow-up
Favelier et al,^10^ 2015	78	F	NR	Azygos vein aneurysm	Endovascular exclusion of azygos vein aneurysm	Covered stent	No postoperative events
Cronan et al,^11^ 2017	15	M	NR	DVT of common iliac veins and IVCA	Stent grafting of azygos vein	14-mm-diameter Zilver stent (Cook Medical Inc); 14-mm Wallstent endoprosthesis (Boston Scientific), 10-mm Wallstent endoprostheses	15 months; uneventful
DeMaio et al,^11^ 2021	28	F	NR	Traumatic blunt injury; mediastinal hematoma	Stent grafting of azygos vein	8-mm × 5-cm Viabahn stent graft (W.L. Gore & Associates)	24 months; uneventful
Present report	70	M	Iatrogenic right iliac vein injury	Recurrent DVT, IVCO, CVI, ulceration without improvement	Stent grafting of azygos vein and left femoral vein	Three 14-mm Wallstent (Boston Scientific)	12 years; uneventful
Present report	51	M	CVI, IVCA	IVCA, recurrent DVT, IVCO, CVI, ulceration without improvement	Stent grafting of azygos vein	14-mm Zilver stent (Cook Medical Inc)	3 months; uneventful

*CVI,* Chronic venous insufficiency; *DVT,* deep vein thrombosis; *F,* female; IVCA, inferior vena cava atresia; *IVCO,* inferior vena cava occlusion; *M,* male.

**Table II:** Clinical scores showing clinical disease burden and improvement after treatment

Pt. No.	Affected leg	Preoperative score		Postoperative score	
		CEAP class C	VCSS	CEAP class C	VCSS
1	RLE	6	20	5^a^	6^a^
	LLE	6	21	5^a^	7^a^
2	RLE	6	19	5^b^	7^b^

*CEAP,* Clinical, Etiology, Anatomy, Pathophysiology; *LLE,* left lower extremity; *Pt. No.,* patient number; *RLE,* right lower extremity; *VCSS,* venous clinical severity score.

a Score after 12 years of follow-up.

b Score 6 months after the procedure.

## Conclusions

Endovascular AVS is rarely performed for iliocaval revascularization. Although previous descriptions are scarce, using the azygos vein for venous outflow can be an effective alternative when other options are not feasible.

## Disclosures

None
